# Intravital Imaging Reveals Divergent Cytokine and Cellular Immune Responses to Candida albicans and Candida parapsilosis

**DOI:** 10.1128/mBio.00266-19

**Published:** 2019-05-14

**Authors:** Linda S. Archambault, Dominika Trzilova, Sara Gonia, Cheryl Gale, Robert T. Wheeler

**Affiliations:** a Department of Molecular and Biomedical Sciences, University of Maine, Orono, Maine, USA; b Department of Pediatrics, University of Minnesota, Minneapolis, Minnesota, USA; c Graduate School of Biomedical Sciences, University of Maine, Orono, Maine, USA; University of Szeged; Leibniz Institute for Natural Product Research and Infection Biology-Hans Knoell Institute Jena (HKI)

**Keywords:** Candida albicans, Candida parapsilosis, cytokine, epithelial cells, innate immunity, intravital imaging, mucosal immunity, phagocyte

## Abstract

*Candida* yeasts are common commensals that can cause mucosal disease and life-threatening systemic infections. While many of the components required for defense against Candida albicans infection are well established, questions remain about how various host cells at mucosal sites assess threats and coordinate defenses to prevent normally commensal organisms from becoming pathogenic. Using two *Candida* species, C. albicans and C. parapsilosis, which differ in their abilities to damage epithelial tissues, we used traditional methods (pathogen CFU, host survival, and host cytokine expression) combined with high-resolution intravital imaging of transparent zebrafish larvae to illuminate host-pathogen interactions at the cellular level in the complex environment of a mucosal infection. In zebrafish, C. albicans grows as both yeast and epithelium-damaging filaments, activates the NF-κB pathway, evokes proinflammatory cytokines, and causes the recruitment of phagocytic immune cells. On the other hand, C. parapsilosis remains in yeast morphology and elicits the recruitment of phagocytes without inducing inflammation. High-resolution mapping of phagocyte-*Candida* interactions at the infection site revealed that neutrophils and macrophages attack both *Candida* species, regardless of the cytokine environment. Time-lapse monitoring of single-cell gene expression in transgenic reporter zebrafish revealed a partitioning of the immune response during C. albicans infection: the transcription factor NF-κB is activated largely in cells of the swimbladder epithelium, while the proinflammatory cytokine tumor necrosis factor alpha (TNF-α) is expressed in motile cells, mainly macrophages. Our results point to different host strategies for combatting pathogenic *Candida* species and separate signaling roles for host cell types.

## Abstract (precis)

In modern medicine, physicians are frequently forced to balance immune suppression against immune stimulation to treat patients such as those undergoing transplants and chemotherapy. More-targeted therapies designed to preserve immunity and prevent opportunistic fungal infection in these patients could be informed by an understanding of how fungi interact with professional and nonprofessional immune cells in mucosal candidiasis. In this study, we intravitally imaged these host-pathogen dynamics during *Candida* infection in a transparent vertebrate model host, the zebrafish. Single-cell imaging revealed an unexpected partitioning of the inflammatory response between phagocytes and epithelial cells. Surprisingly, we found that *in vivo* cytokine profiles more closely match *in vitro* responses of epithelial cells rather than phagocytes. Furthermore, we identified a disconnect between canonical inflammatory cytokine production and phagocyte recruitment to the site of infection, implicating noncytokine chemoattractants. Our study contributes to a new appreciation for the specialization and cross talk among cell types during mucosal infection.

## INTRODUCTION

Fungal species of the genus *Candida* are commensals on mucosal surfaces in healthy human hosts but cause both invasive and mucosal candidiasis when immune defenses are compromised (1, 2). While Candida albicans is the species most commonly isolated from patients, infections due to C. parapsilosis are increasing, especially in neonates born prematurely (3–5). In healthy hosts, *Candida* is maintained as a commensal through the defenses of professional immune cells and the barrier functions of the mucosal epithelium. When these defenses are compromised, mucosal candidiasis ensues (1, 6). Understanding how host cells at mucosal surfaces interact with fungal cells and how they coordinate their antifungal defenses will inform our attempts to prevent both systemic and mucosal disease (7, 8).

The mucosal epithelium is a complex environment, and protection from mucosal candidiasis requires the combined actions of several cell types. In addition to their barrier functions, epithelial cells respond to *Candida* by inhibiting *Candida* growth with antimicrobial peptides and recruiting immune effector cells with alarmins and proinflammatory cytokines (9–12). Among immune cells, neutrophils play key roles in defense at mucosal surfaces and in preventing dissemination of C. albicans (13, 14). *In vitro*, neutrophil/epithelial cross talk provides protection from C. albicans (15–17). However, neutrophil activity must be tightly controlled, as evidenced by its role in worsening symptoms of vulvovaginal candidiasis (18–20). Monocytes/macrophages are essential for establishing protective immunity to disseminated infection, but their role in mucosal infection is not completely clear (21–25). Evidence from mouse and zebrafish models points to the redundancy of macrophages in mucosal C. albicans infections (26, 27). However, macrophages have been shown to protect against other fungi in mucosal infection (28–31). C. parapsilosis is known to interact with macrophages and monocytes *in vitro*, but the roles of phagocytes in controlling C. parapsilosis infection have not yet been explored in any live vertebrate infection model.

Epithelial cells and patrolling phagocytes are the first host cells to detect pathogens and signal to coordinate defenses against mucosal candidiasis (6, 32, 33). *In vitro* experiments with single cell types have shown that epithelial cells and phagocytes differ with respect to inflammatory signaling during challenge by C. albicans and C. parapsilosis. Epithelial cells from oral and intestinal sources (the oral cell lines SCC15 and TR146 and the primary human enterocyte cell line H4) respond *in vitro* to C. albicans by producing proinflammatory cytokines but produce little cytokine response to C. parapsilosis (15, 34, 35). On the other hand, professional innate immune cells, including human peripheral blood mononuclear cells, murine peritoneal macrophages, and the murine macrophage cell line J774.2, produce proinflammatory cytokines in response to both heat-killed C. albicans and C. parapsilosis (36–38). These contradictory results make it difficult to predict how the different cell types in mucosal tissues coordinate defense against these opportunistic fungal pathogens, so we sought to measure immune responses in a tractable vertebrate mucosal infection model.

*In vitro* experiments are limited to a few host cell types, and *in vivo* imaging in mammalian models is technically difficult (39–41). Complex signaling interactions between different host cell populations during mucosal Candida albicans infection were hinted at in studies using *in vitro* models with two or more host cell types (16, 17) and have been further elucidated using fluorescence-activated cell sorting of infected mouse tissue (9, 42, 43). Although these studies have shed light on the signaling roles and interactions of various host cell types with C. albicans, there remain significant gaps in our knowledge about the dynamics and cell type specificity of immune responses in the host, especially with respect to infections with other clinically important *Candida* species, such as C. parapsilosis. To further explore these *in vitro* and *in vivo* findings using intravital imaging, we turned to the zebrafish swimbladder mucosal model, which mimics many aspects of mammalian infection (27, 44). The swimbladder is a natural site of fungal infection initiation in the fish that shares functional, anatomical, ontological, and transcriptional similarities to the lung (45–54). We compared the mucosal immune responses to two clinically relevant *Candida* species in an environment containing multiple host cell types, measuring several aspects of the immune response, including pathway activation, cytokine production, and innate immune recruitment. While C. albicans activated nuclear factor kappa B (NF-κB) signaling and elicited a strong proinflammatory cytokine response at this mucosal site, the host inflammatory response to C. parapsilosis was muted, similar to what has been found *in vitro* for epithelial cells. Live single-cell imaging suggests that NF-κB activation and tumor necrosis factor alpha (TNF-α) upregulation occur in different cellular subsets. Interestingly, the inflammatory cytokine response was not predictive of phagocyte behavior, as neutrophils and macrophages were recruited to and attacked both *Candida* species. Nevertheless, neutrophils were essential for protection from only C. albicans and not C. parapsilosis, confirming their known role in attacking hyphae. The differential immune responses to the two species reveal a disconnection between chemokine production and phagocyte recruitment. Single-cell intravital imaging further suggests that there is tissue-specific activation of NF-κB and TNF-α expression in mucosal candidiasis.

## RESULTS

### C. albicans causes lethal infection, but C. parapsilosis does not.

**FIG 1:**
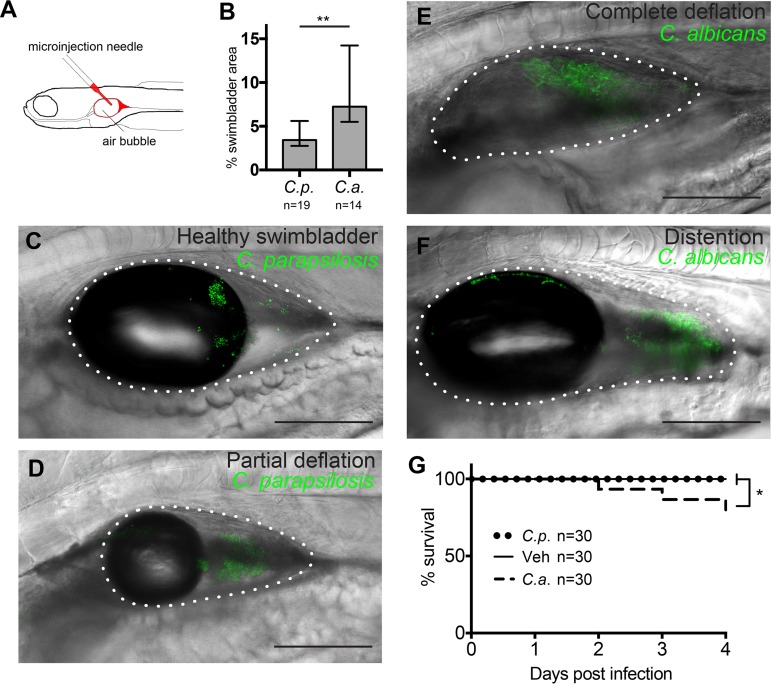
C. albicans (*C.a.*) is more virulent than C. parapsilosis (*C.p.*) in the zebrafish swimbladder infection model. (A) Zebrafish were infected in the swimbladder at 4 days postfertilization (dpf) with 50 to 100 yeast cells. (B) *Candida* burden at 24 h postinfection (hpi) quantified from confocal z-projections. Data were pooled from 4 experiments. (C to F) Examples of infected swimbladders in *Tg*(*mpx*:*mCherry*):*uwm7Tg* zebrafish infected with C. parapsilosis (C and D) or C. albicans (E and F). Depicted are normal appearance of swimbladder (6 hpi) (C), partial swimbladder deflation (24 hpi) (D), complete deflation (24 hpi) (E), and distended swimbladder (24 hpi) (F). Bars, 150 μm. The dotted white line indicates the boundary of the swimbladder. (G) Injected fish were monitored for survival for 4 dpi. Data were pooled from 3 independent experiments. Statistics are described in Materials and Methods (*, *P ≤ *0.05; **, *P ≤ *0.01). Veh, vehicle.

C. parapsilosis and C. albicans are opportunistic pathogens that live commensally on mucosal surfaces of healthy humans and elicit different reactions from immune and epithelial cells *in vitro* (34, 35). To explore the relative virulence of these two fungal species in the mucosal setting in a live vertebrate host, we modified the zebrafish swimbladder infection model previously developed in our laboratory (27, 44, 55). We performed infection with a larger inoculum of 50 to 100 yeast cells to promote morbidity without immunocompromising the host (Fig. 1A). Both *Candida* species grew readily in the swimbladder, with C. albicans covering about twice as much area as C. parapsilosis by 24 h postinfection (hpi) (Fig. 1B). In the high-inoculum infection of immunocompetent fish used in this study, the swimbladder remained fully inflated and appeared healthy in the first hours after infection (Fig. 1C). However, within 24 hpi, signs of disease were apparent, with swimbladders becoming partially (Fig. 1D) or completely (Fig. 1E) deflated. Over time, the swimbladder could become greatly distended (Fig. 1F), and in C. albicans infections, hyphae sometimes breached the swimbladder epithelium, a factor predictive of fish death (27, 56). C. parapsilosis infection caused no mortality within 4 days postinfection (dpi), while C. albicans-infected animals began to perish at 2 dpi and reached 20% mortality by 4 dpi (Fig. 1G). Thus, in these high-inoculum infections, only C. albicans caused patterns of disease leading to mortality that were similar to those previously seen in immunocompromised fish and in a mixed fungal-bacterial infection (27, 56).

### Zebrafish infected with C. albicans produce higher levels of inflammatory cytokines than C. parapsilosis-infected fish.

**FIG 2:**
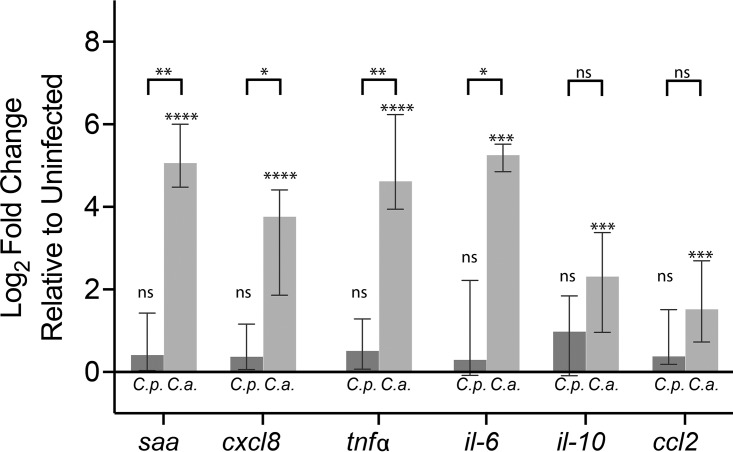
C. albicans elicits higher levels of cytokine expression than C. parapsilosis. Zebrafish were infected at 4 dpf as described in the legend of Fig. 1. At 24 hpi, total RNA was extracted from groups of 9 to 14 fish. Gene expression levels were determined by qPCR relative to mock-infected fish using the 2^−ΔΔ*C_T_*^ method. Data are from 11 independent experiments. Notations above each bar indicate the significance of the difference between experimental treatments and vehicle-injected controls. Notations above the brackets indicate if there was a difference between C. parapsilosis*-* and C. albicans-infected fish. Statistics are described in Materials and Methods (*, *P < *0.05; **, *P < *0.01; ***, *P < *0.001; ****, *P < *0.0001; ns, not significant [*P > *0.05]). Abbreviations: *saa*, serum amyloid A gene; *tnf*α, tumor necrosis factor alpha gene; *il-10*, interleukin-10 gene; *ccl2*, C-C motif chemokine ligand 2 gene; *cxcl8*, C-X-C motif ligand 8 gene; *il-6*, interleukin-6 gene.

Because we saw differences in the severity of the infections, we expected to find different inflammatory responses to the two *Candida* species. We measured changes in the expression of six inflammation-associated cytokines at 24 hpi (Fig. 2). In C. albicans infection, expression was significantly elevated above control levels for all 6 cytokines and higher than that observed in C. parapsilosis infection for 4 of 6 cytokines. In contrast, in C. parapsilosis-infected fish, the median levels of cytokine expression were not significantly elevated above controls. Thus, C. albicans evokes a stronger whole-fish cytokine response than C. parapsilosis during *in vivo* mucosal infection, demonstrating that there are important differences in the immune response at this early time point, hours before mortality is observed.

### The local inflammatory signaling pattern mirrors whole-fish cytokine levels.

The whole-fish quantitative PCR (qPCR) data showed overall cytokine responses but did not give us any spatial information about inflammatory signaling or indicate the cell types involved. In the zebrafish, local immune activation and cytokine signaling by epithelial tissue and innate immune cells can be imaged in real time in the live host. Two key signaling components activated by *Candida* are NF-κB and TNF-α (44, 57–61). TNF-α expression is activated downstream of NF-κB and other signaling pathways and is implicated in protective cross talk between polymorphonuclear cells and the oral epithelium (17, 62).

**FIG 3:**
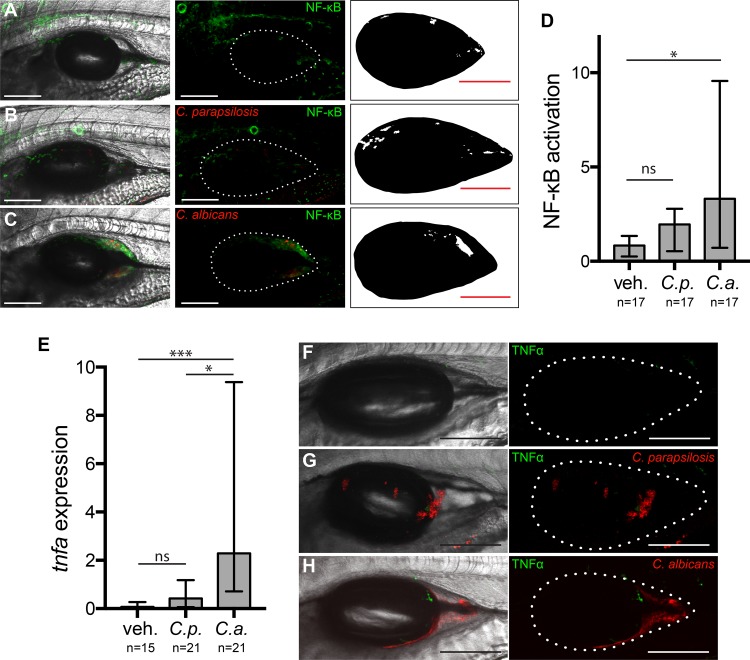
Transcription factor NF-κB is activated and proinflammatory cytokine TNF-α is expressed during C. albicans but not C. parapsilosis infection. Transgenic *Tg*(*NF-*κ*B*:*EGFP*) zebrafish were infected and imaged as described in the legend of Fig. 1. (A to C) Images representing the median levels of NF-κB activation for vehicle (A), C. parapsilosis (B), and C. albicans (C) injections. Panels A to C show maximum projections of 12 z-slices. (Left) Overlay of fluorescence and differential interference contrast (DIC); (middle) overlay of fluorescence with a dotted outline of the swimbladder; (right) thresholded image for quantification. (D) Quantification of NF-κB activation. Data are from 3 independent experiments. (E to H) *TgBAC*(*tnfa*:*GFP*) reporter fish were infected and imaged at 24 hpi as described above. (E) Quantification of TNF-α expression. Data are from 3 independent experiments. (F to H) Representative images of swimbladders. Median levels of TNF-α expression are shown for the vehicle control (F) and C. parapsilosis (G) and C. albicans (H) infections. (Left) Maximum projections of 15 to 18 z-slices; (right) dotted outline of swimbladder. All bars, 150 μm. Statistics are described in Materials and Methods (*, *P ≤ *0.05; **, *P ≤ *0.01; ***, *P ≤ *0.001; ns, not significant [*P > *0.05]).

To detect activation of NF-κB at the infection site, we used a transgenic zebrafish line, *Tg*(*NF-*κ*B*:*EGFP*), that reports on pathway activity in multiple cell types and is activated in the swimbladder upon oral infection (44, 63). [The current zebrafish genetic nomenclature uses colons to indicate the following organization for transgenic fish lines: *Tg*(regulatory sequence:coding sequence).] Imaging of infected fish at 24 hpi revealed significant induction of NF-κB in C. albicans-infected fish but only basal levels of activity in C. parapsilosis-infected fish (Fig. 3A to D). As expected, we found green fluorescent protein (GFP) expression in several tissues, but not the swimbladder, under homeostatic conditions (63). To visualize local cytokine expression, we used *TgBAC*(*tnfa*:*GFP*) reporter fish (64). Again, we saw significant activation of *tnfa*:*GFP* in only C. albicans and not C. parapsilosis infections (Fig. 3E to G).

Intriguingly, despite the well-characterized connections between NF-κB and TNF-α, our *in vivo* imaging revealed differences in the spatial patterns of NF-κB activation and expression of TNF-α during C. albicans infection. NF-κB:EGFP fluorescence was more diffuse (Fig. 3C), while *tnfa*:*GFP* expression was more punctate and visible mainly near C. albicans yeast and hyphae (Fig. 3H). These patterns of activity were especially interesting because previous work has shown that, in addition to the resident phagocytes present without infection, recruited phagocytes are present within the epithelium-lined swimbladder at this time postinfection (27, 44, 56) (see below).

### Signaling patterns differ in macrophages and epithelial tissue.

**FIG 4:**
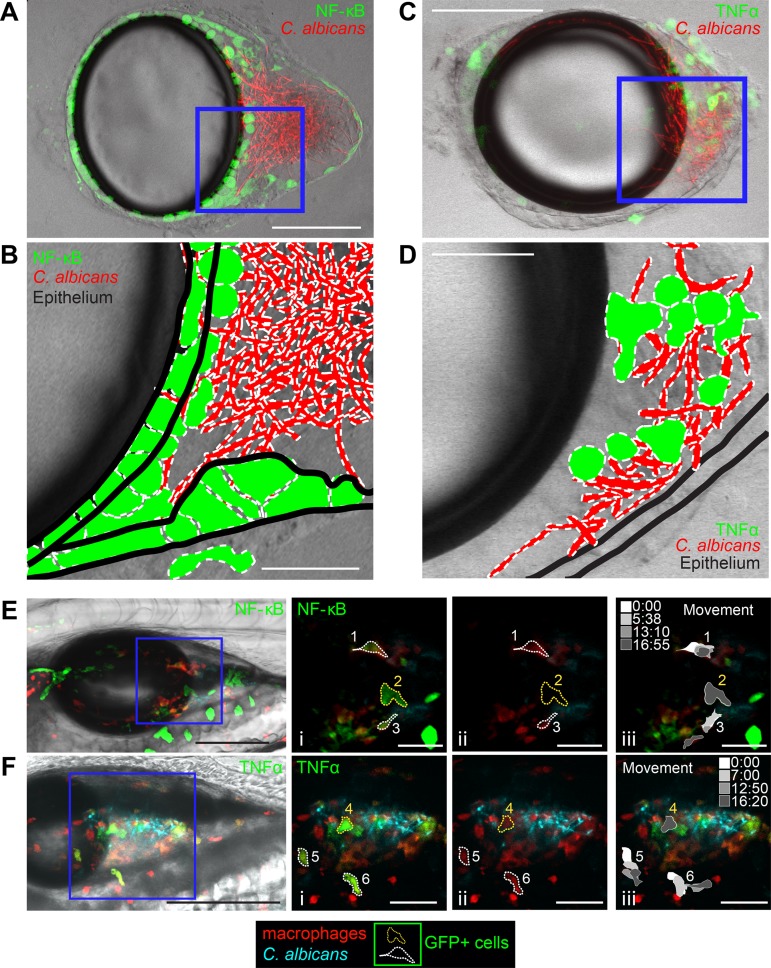
Patterns of NF-κB activation and TNF-α expression differ. Dissected swimbladders from C. albicans-infected fish were imaged at 24 hpi. (A) z-projection of 3 slices of a dissected *Tg*(*NF-*κ*B*:*EGFP*) swimbladder with moderate EGFP expression. (B) Single z-slice from the blue square in the z-stack in panel A, with outlines of fungi, EGFP^+^ cells, and epithelial layers based on the DIC image. (C) z-projection of 7 slices of a *TgBAC*(*tnfa*:*GFP*) swimbladder with high GFP expression levels. (D) Single z-slice from the blue square in the z-stack in panel A, with outlines of fungi, GFP^+^ cells, and epithelial layers based on the DIC image. (E and F) Still images from time-lapse images taken at 24 hpi. (E) *Tg*(*NF-*κ*B*:*EGFP*) × *mpeg1*:*dTomato* (red macrophage) zebrafish at time 0:00 of the time-lapse image in Movie S1 in the supplemental material. The leftmost image is a maximum-projection overlay of all colors using a middle plane from the DIC image. (i) Zoomed-in images of the areas outlined in the blue square. Dotted lines outline example cells that either moved (white outlines [cells 1 and 3]) or remained stationary (yellow outlines [cell 2]) over the 16-min-long time-lapse experiment. (ii) The GFP channel was eliminated to demonstrate red fluorescence of macrophages. Cells 1 and 3 are dTomato^+^ (macrophages), while cell 2 is not. (iii) Schematics showing the positions of each cell at the times indicated in the grayscale legend. Only cells 1 and 3 change shape or position. (F) *TgBAC*(*tnfa*:*GFP*) × *mpeg1*:*dTomato* zebrafish at time 0:00 of the time-lapse imaging in Movie S2. (i) Outlines of example cells (white, moved [cells 5 and 6]; yellow, stationary [cell 4]). (ii) Cells 4, 5, and 6 are dTomato^+^ (macrophages). (iii) Schematics showing movement over time. Cells 5 and 6 change shape and position over the course of the time-lapse experiment, but cell 4 does not. Color channels show z-projections of 13 slices (E) or 11 slices (F). DIC was performed for a single z-slice. Bars, 150 μm (A, C, E, and F) and 50 μm (B, D, Ei to Eiii, and Fi to Fiii).

While live imaging of transgenic fish at low resolution narrowed the location of signaling to the infection site, it did not allow us to identify which cell types were activated and contributing to swimbladder fluorescence. Because of the differences in NF-κB and TNF-α patterns, we reasoned that the two signaling components might be activated in different cell types. To examine cellular expression at high resolution and distinguish between fluorescence within the swimbladder and fluorescence in overlying tissue, we dissected swimbladders from C. albicans-infected zebrafish using a method previously developed in our laboratory (55). Imaging of *Tg*(*NF-*κ*B*:*EGFP*) zebrafish swimbladders immediately after dissection revealed GFP-positive (GFP^+^) cells of the epithelial layer both near and distant from the area at the back of the swimbladder containing fungi (Fig. 4A and B). This is also illustrated in a single representative slice by outlining fluorescent cells and adding tissue landmarks (Fig. 4B). In *TgBAC*(*tnfa*:*GFP*) zebrafish, GFP-positive cells were not seen in the epithelial layer, but many GFP-positive cells were intermingled with yeast and hyphae (Fig. 4C and D). This is again illustrated in a representative z-slice (Fig. 4D). The morphology and location of these cells are consistent with those of phagocytes.

10.1128/mBio.00266-19.5 MOVIE S1 Time-lapse movie made from 10 consecutive images (magnification, ×10) of a representative C. albicans-infected *Tg*(*NF-*κ*B*:*EGFP*) × *mpeg1*:*dTomato* zebrafish swimbladder at 26 hpi. Shown is the maximum projection of 13 z-slices. Bar, 100 μm. Red, macrophages; green, *NF-*κ*B*:*EGFP*-positive cells; cyan, C. albicans. The white outline indicates the boundary of the swimbladder. The total time was 16 min 55 s. Download Movie S1, AVI file, 15.7 MB. Copyright © 2019 Archambault et al. 2019 Archambault et al. This content is distributed under the terms of the Creative Commons Attribution 4.0 International license.

10.1128/mBio.00266-19.6 MOVIE S2 Time-lapse movie made from 28 consecutive images (magnification, ×20) of a C. albicans-infected *TgBAC*(*tnfa*:*GFP*) swimbladder with high GFP expression levels at 26 hpi. Shown is the maximum projection of 11 z-slices. Bar, 100 μm. Red, macrophages; green, *tnfa*:*GFP-*positive cells; cyan, C. albicans. The white outline indicates the boundary of the swimbladder. The total time was 32 min 39 s. Download Movie S2, AVI file, 0.5 MB. Copyright © 2019 Archambault et al. 2019 Archambault et al. This content is distributed under the terms of the Creative Commons Attribution 4.0 International license.

To further characterize these cells displaying immune activation, we assessed their motility by crossing *Tg*(*NF-*κ*B*:*EGFP*) or *TgBAC*(*tnfa*:*GFP*) fish with *mpeg1*:*dTomato* (red macrophage [65]) reporter fish and using time-lapse imaging to view the shape, behavior, and identity of GFP-fluorescing cells in infected fish. We found in time-lapse experiments that *mpeg1*:*dTomato*^+^ macrophages were occasionally doubly positive for *NF-*κ*B*:*EGFP* or *tnfa*:*GFP* (6/43 for *NF-*κ*B*:*EGFP* and 7/35 for *tnfa*:*GFP*) (Fig. 4E and F; see also Movies S1 and S2 in the supplemental material). Cells that are GFP^+^ are outlined and were monitored for more than 16 min (Fig. 4Ei to Eiii and Fig. 4Fi to Fiii). In *TgBAC*(*tnfa*:*GFP*) fish, all GFP^+^ cells (7/7) were also dTomato^+^, indicating that they are macrophages, while this was the case for only a minority of GFP^+^ cells in *Tg*(*NF-*κ*B*:*EGFP*) fish (5/57) (Fig. 4Eii and Fig. 4Fii). Many GFP^+^ cells were motile in *tnfa*:*GFP* transgenic fish (5/7), but only a few were motile in *NF-*κ*B*:*EGFP* transgenic fish (3/57) (Fig. 4Eiii and Fig. 4Fiii). This indicates that while TNF-α expression in the swimbladder is limited to macrophages, NF-κB signaling is activated in both macrophages and other cells likely to be epithelial.

Large, nonmotile cells in *Tg*(*NF-*κ*B*:*EGFP*) fish, such as cell 2 (Fig. 4Eiii, yellow dotted outline), were enhanced green fluorescent protein positive (EGFP^+^) but dTomato negative (dTomato^−^), suggesting that they are not macrophages. In fact, the position and behavior of such cells suggest that they reside in the swimbladder epithelial layer, consistent with what is observed in dissected swimbladders (Fig. 4A and B). In *TgBAC*(*tnfa*:*GFP*) fish, some stationary cells, such as cell 4 in the time-lapse image (Fig. 4Fiii, yellow dotted outline), were interacting with *Candida* and were identified as macrophages based on their *mpeg1*:*dTomato* expression. These time-lapse data thus indicate that TNF-α-expressing cells are more likely to be motile macrophages, while NF-κB is most frequently activated in nonmotile cells with epithelial morphology.

### Neutrophils are recruited to infection and attack both C. albicans and C. parapsilosis.

**FIG 5:**
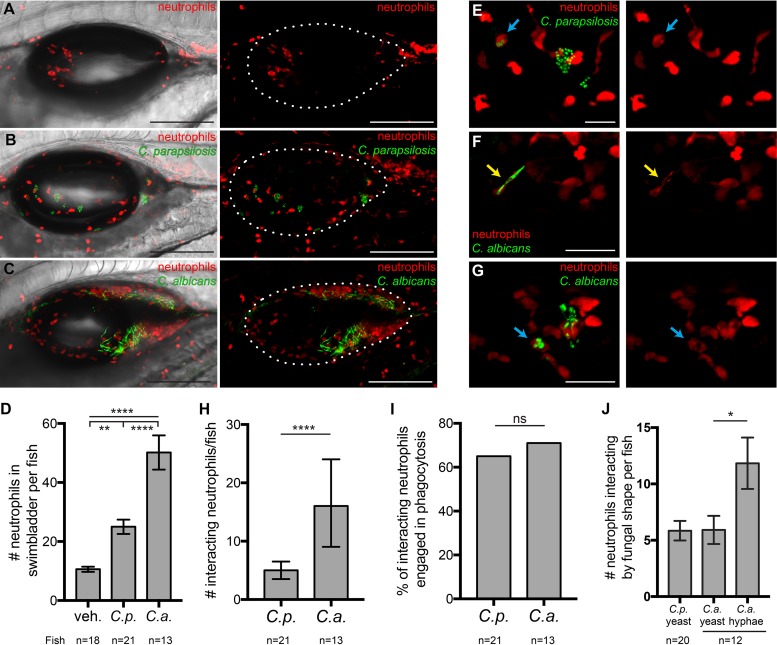
Neutrophils respond to infections with both *Candida* species. *Tg*(*mpx*:*mCherry*):*uwm7Tg* zebrafish (red neutrophils) were infected as described in the legend of Fig. 1 and imaged at 24 hpi. Data are pooled from 5 independent experiments. (A to C) Representative images from vehicle (A), C. parapsilosis (B), and C. albicans (C) cohorts. Maximum projections of 19 z-slices (A), 18 z-slices (B), and 16 z-slices (C), with (left) and without (right) a single DIC z-slice, are shown. (D) Neutrophils per fish in the swimbladder lumen at 24 hpi. (E to G) Examples of neutrophils (red) interacting with C. parapsilosis (green) (E) or C. albicans (green) (F and G). Interactions include contact, phagocytosis (E and G, blue arrows), and “frustrated phagocytosis” (F, yellow arrows). Maximum projections of 3 slices (E and F) and 9 slices (G) are shown. (H) Numbers of neutrophils per fish involved in interactions with C. parapsilosis or C. albicans at 24 hpi. (I) Percentages of interacting neutrophils engaged in phagocytosis at 24 hpi. (J) Numbers of neutrophils per fish interacting with yeast of C. parapsilosis and yeast or hyphae of C. albicans. Numbers of neutrophils scored for the vehicle, C. parapsilosis, and C. albicans were 191, 525, and 652, respectively. Statistics are described in Materials and Methods (*, *P ≤ *0.05; **, *P ≤ *0.01; ***, *P ≤ *0.001; ****, *P ≤ *0.0001; ns, not significant [*P > *0.05]). Bars, 150 μm (A to C) and 40 μm (E to G).

The activation of NF-κB and expression of TNF-α at the infection site in C. albicans-infected fish, combined with the qPCR data showing that the chemokines CXCL8 and CCL2 were upregulated only in C. albicans infection, suggested that phagocytes might be recruited only to C. albicans infections. We measured neutrophil recruitment using the *Tg*(*mpx*:*mCherry*)*uwm7Tg* fish line, which has been characterized to express red fluorescence almost exclusively in neutrophils (66). To our surprise, we found increased neutrophil recruitment compared to mock infections (11 neutrophils/fish) for both C. parapsilosis (25/fish) and C. albicans (50/fish) infections (Fig. 5A to D).

Because of the different cytokine milieus elicited by the two fungal species, we reasoned that there might be differential interactions of neutrophils with each fungal species at the infection site. We examined z-stack images slice-by-slice and catalogued interactions between neutrophils and *Candida* (Fig. 5E to G). In C. albicans infection, significantly more neutrophils per fish were involved in interactions with the fungus, although this is not surprising considering their greater numbers in C. albicans-infected swimbladders (Fig. 5H). Interactions in which neutrophils had ingested C. parapsilosis (Fig. 5E, blue arrows) or C. albicans (Fig. 5G, blue arrows) yeast cells or were wrapped around C. albicans hyphae (“frustrated phagocytosis”) (Fig. 5F, yellow arrows) were counted as phagocytosis. When all neutrophils interacting with *Candida* were considered together, similar percentages were engaged in phagocytosis in C. parapsilosis (∼65%) and C. albicans (∼72%) infections (Fig. 5I). Thus, despite the lower numbers of neutrophils in C. parapsilosis infection and the differing cytokine environment, neutrophils had similar levels of activity against each fungal species.

Dimorphic switching of C. albicans is considered an important virulence trait, although little is known about how different morphotypes interact with immune cells *in vivo*. In the swimbladder, C. albicans injected as yeast switches rapidly to hyphal growth within the first 3 hpi (55, 56), and here we found that C. parapsilosis remains in the yeast form throughout the infection period. Neutrophils were found interacting more often with C. albicans hyphae than with yeast, which could be due to the large number of hyphal segments present (Fig. 5J). Overall, these data are consistent with the known activities of neutrophils against C. albicans hyphae and yeast *in vitro* (67–70). In summary, neutrophils are recruited to and actively interact with fungal cells of both *Candida* species, despite the nearly undetectable levels of inflammatory cytokine production in C. parapsilosis infection.

### Macrophages are recruited to infections with both Candida species.

**FIG 6:**
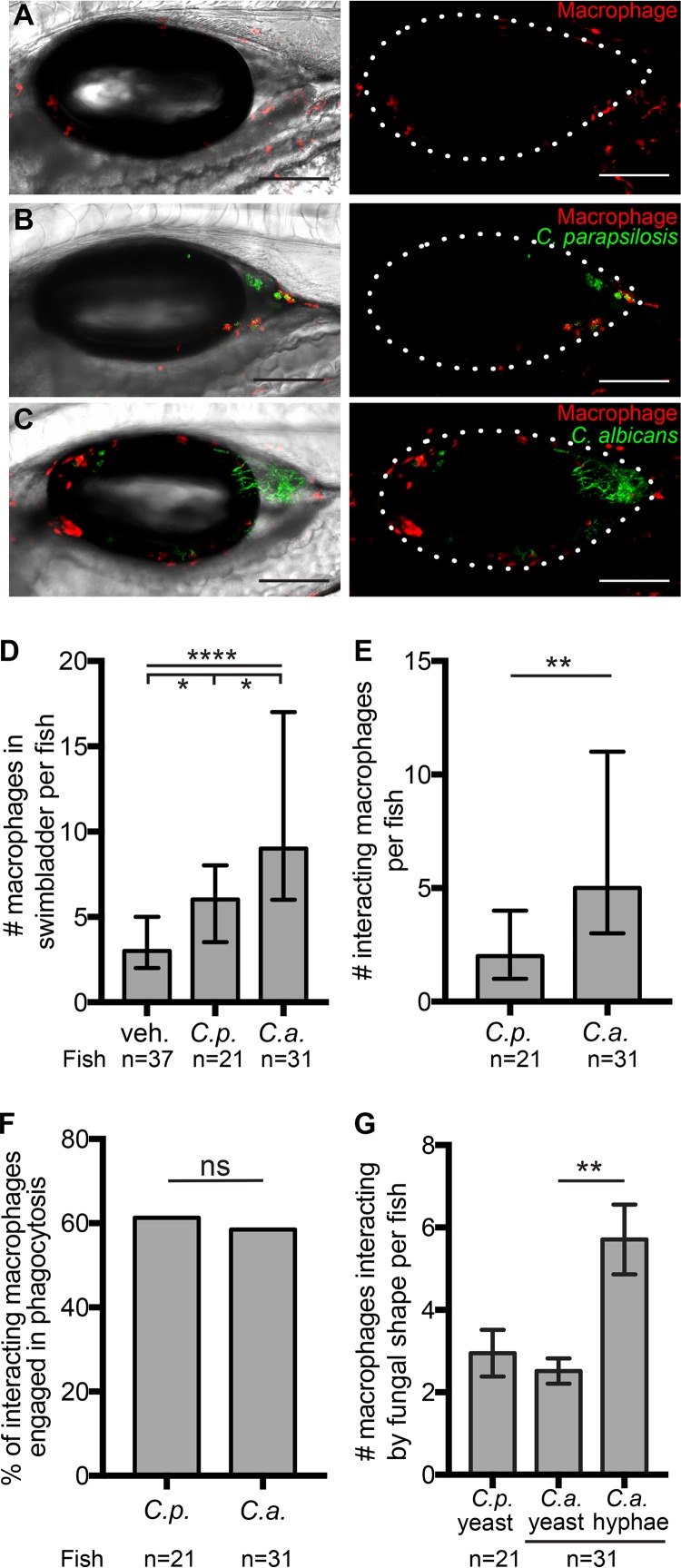
Both C. albicans and C. parapsilosis elicit macrophage recruitment. Transgenic *mpeg1*:*GAL4/UAS*:*nfsB-mCherry* zebrafish (red macrophages) were infected and imaged at 24 hpi. (A to C) Representative images of zebrafish swimbladders injected with the vehicle (A), C. parapsilosis (B), and C. albicans (C). Maximum projections of 16 slices (A) and 13 slices (B and C), with (left) and without (right) a single DIC z-slice, are shown. (D) Numbers of macrophages per fish in the swimbladder lumen. Data were pooled from 7 independent experiments. (E) Numbers of macrophages per fish involved in interactions with C. parapsilosis or C. albicans. (F) Percentages of interacting macrophages engaged in phagocytosis. (G) Numbers of macrophages per fish interacting with fungi. Numbers of macrophages scored for the vehicle, C. parapsilosis, and C. albicans were 137, 135, and 367, respectively. Statistics are described in Materials and Methods (*, *P ≤ *0.05; **, *P ≤ *0.01; ***, *P ≤ *0.001; ****, *P ≤ *0.0001; ns, not significant [*P > *0.05]). Bars, 150 μm (A to C).

Although patrolling macrophages play an important role in the initiation of inflammation through the production of cytokines and are essential for controlling invasive candidiasis, they are thought to play a redundant role in mucosal *Candida* infection (23, 26, 27, 71–74). Nevertheless, we observed a significant C. albicans-specific induction of *ccl2*, which suggested that macrophages would be recruited only upon C. albicans infection. To our surprise, we found increased numbers of macrophages in the swimbladders of both C. parapsilosis-infected and C. albicans-infected fish (medians of 3 macrophages for mock-infected fish, 6 for C. parapsilosis-infected fish, and 9 for C. albicans-infected fish) (Fig. 6A to D).

Patterns of macrophage interaction with *Candida* cells were remarkably similar to those of neutrophils. We found more macrophages interacting with the pathogen in C. albicans infections (median of 5 macrophages per fish) than in C. parapsilosis infections (median of 2 per fish) (Fig. 6E). As was the case for neutrophils, similar percentages (around 60%) of macrophages interacting with the two pathogens were engaged in phagocytosing them (Fig. 6F). Macrophages, like neutrophils, were found interacting with C. albicans hyphae more often than with yeast (Fig. 6G). Thus, macrophages are recruited to infections with both *Candida* species, and although they are found in lower numbers than neutrophils, they interact with and phagocytose both species.

### Functional neutrophils are required for protection from C. albicans but not C. parapsilosis infection.

**FIG 7:**
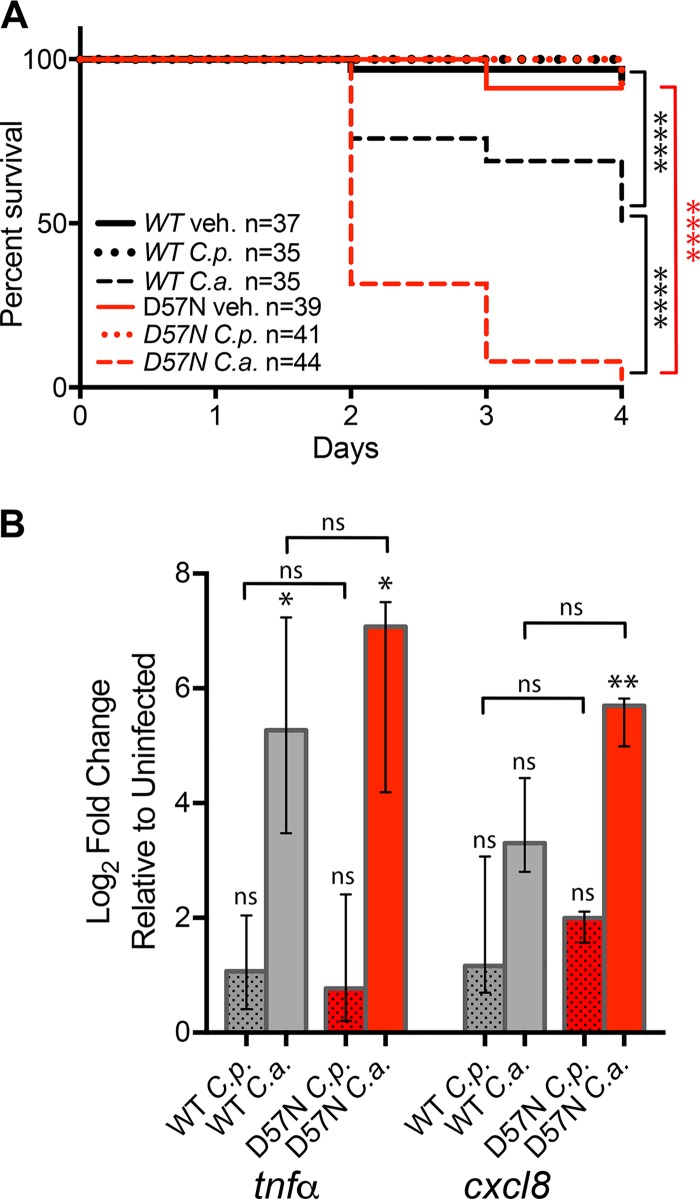
Neutrophil defects impact immunity to C. albicans but not C. parapsilosis infection. (A) *Tg*(*mpx*:*mCherry-2A-Rac2D57N*) (D57N) zebrafish and their wild-type (WT) siblings were infected at 4 dpf and monitored for 4 days. Survival curves are based on data pooled from 3 independent experiments. (B) qPCRs of cohorts of 10 fish, in 3 independent experiments, performed as described in the legend of Fig. 2. The median log_2_-fold changes relative to vehicle-injected fish are plotted. Gray bars, WT; red bars, D57N mutant; dotted bars, C. parapsilosis-infected fish; solid bars, C. albicans-infected fish. Notations above individual bars indicate differences between *Candida*-infected and vehicle-injected groups. Notations above brackets indicate differences between WT and D57N fish. Statistics are described in Materials and Methods (*, *P ≤ *0.05; **, *P ≤ *0.01; ***, *P ≤ *0.001; ns, not significant [*P > *0.05]).

High levels of neutrophil engagement suggested to us that these cells play an important role in the immune response to both *Candida* species in the swimbladder model. We were interested to see if neutrophilic inflammation is protective, as in the murine oral infection models, or damaging, as in human vulvovaginal infection (18, 75). To block neutrophil function, we employed the transgenic fish line *Tg*(*mpx*:*mCherry-2A-Rac2D57N*) (D57N), a model of leukocyte adhesion deficiency in which neutrophils are present but defective in extravasation and phagocytosis (76–79). In the low-dose swimbladder infection model, neutrophils in D57N zebrafish fail to migrate into the C. albicans-infected swimbladder, and this makes the fish susceptible to invasive disease (27). When infected with higher doses of C. albicans, D57N zebrafish exhibited nearly 100% mortality by 4 dpi, compared to only 50% mortality in their wild-type (WT) siblings (Fig. 7A). Surprisingly, survival rates for D57N fish infected with C. parapsilosis were not significantly different from the nearly 100% survival found in their WT siblings, despite the lack of neutrophil recruitment that was expected in this fish line (Fig. 7A and Movie S3). C. albicans-infected D57N fish had more-severe infections than their WT siblings, with extensive growth of filaments that often breached the swimbladder epithelium.

10.1128/mBio.00266-19.7 MOVIE S3 Three-channel composite movie made from z-series of 21 slices with an interslice interval of 5 μm. This C. parapsilosis-infected Rac2-D57N fish was imaged at 24 hpi. Red, neutrophils; green, C. parapsilosis; white line, boundary of the swimbladder. Magnification, ×10. Bar, 300 μm. Download Movie S3, MOV file, 1.7 MB. Copyright © 2019 Archambault et al. 2019 Archambault et al. This content is distributed under the terms of the Creative Commons Attribution 4.0 International license.

We reasoned that inactivation of neutrophils could alter cytokine signaling through opposing mechanisms: greater damage to epithelial and other tissues could release damage-associated molecular patterns and provoke higher expression levels of inflammatory cytokines, or, alternatively, the absence of neutrophils at the site of infection could eliminate their contribution to amplification of the inflammatory response (80). Surprisingly, we found that D57N fish had nearly identical levels of *tnfa* and *cxcl8* (Fig. 7B) as well as *saa*, *il-10*, and *il-1*β (Fig. S1) expression compared to their WT siblings when infected with C. albicans. Levels of these cytokines were also similar in both WT and D57N infections with C. parapsilosis. These data suggest that neutrophil inactivation does not have a strong overall net effect on inflammatory signaling.

10.1128/mBio.00266-19.1 FIG S1 *Tg*(*mpx*:*mCherry-2A-Rac2D57N*) (D57N) zebrafish and their wild-type (WT) siblings were infected in the swimbladder at 4 days postfertilization (dpf) with 50 to 100 yeast cells. In 3 independent experiments, total RNA was extracted from groups of 10 fish at 24 hpi. Gene expression levels for serum amyloid A (*saa*), interleukin-1β (*il-1*β), and interleukin 10 (*il-10*) relative to mock-infected fish were determined by qPCR using the 2^−ΔΔ*C_T_*^ method. The median log_2_-fold changes relative to the vehicle are plotted. Gray bars, WT; red bars, D57N mutant; dotted bars, C. parapsilosis-infected fish; solid bars, C. albicans-infected fish. Notations above individual bars indicate differences between *Candida*-infected and vehicle-injected groups. Notations above brackets indicate differences between WT and D57N fish. *, *P ≤ *0.05; **, *P ≤ *0.01; ***, *P ≤ *0.001; ns, not significant (*P > *0.05). Download FIG S1, PDF file, 0.7 MB. Copyright © 2019 Archambault et al. 2019 Archambault et al. This content is distributed under the terms of the Creative Commons Attribution 4.0 International license.

## DISCUSSION

Candida albicans and Candida parapsilosis are opportunistic yeast pathogens that live as commensals of healthy people but breach epithelial barriers to cause serious illness in immunocompromised patients. To understand how fungi breach this barrier, it is important to study the interactions between *Candida* cells and host defenses at mucosal surfaces in the intact host. By modeling mucosal *Candida* infection in the transparent larval zebrafish, we were able to visualize interactions between host immune cells, epithelial cells, and fungal pathogens in four dimensions (4D) in the live host. We discovered that mucosal infection by C. albicans, but not C. parapsilosis, caused significant mortality, activated NF-κB signaling, and evoked a strong local proinflammatory response. Despite the differential abilities of the two species to activate inflammatory pathways, infections with both species stimulated the recruitment of neutrophils and macrophages that actively attacked the fungi. Overall, our findings suggest that the contrasting immune responses to the two species of *Candida* in the swimbladder more closely resemble *in vitro* epithelial cell responses than *in vitro* mononuclear phagocyte responses, suggesting an important role for the epithelium in the overall inflammatory response.

The lack of C. parapsilosis virulence in the zebrafish is consistent with what has been seen in other infection models. This is the case for disseminated and mucosal disease in mice (81) as well as *in vitro* challenges with epithelial cells (34, 35, 82). Although C. parapsilosis is a common commensal fungus (5, 83), its virulence is usually associated with the hospital setting, and it is thought that predisposing conditions, such as epithelial damage or barrier breach by medical interventions, lead to disseminated infection (14, 83). In zebrafish models of C. albicans infection, penetrating hyphae are closely associated with mortality, and yeast-locked strains have limited virulence (27, 56, 84). Hyphal growth has also been clearly implicated in epithelial destruction *in vitro* and in mouse disease models (85–88). Thus, while the inability of C. parapsilosis to cause mortality in the absence of neutrophil function may be due to any number of differences between the two species, the lack of filamentous growth and expression of genes coregulated with the hyphal switch (such as candidalysin) are likely to be major determinants of differential virulence (89, 90).

Infection with C. albicans, but not with C. parapsilosis, elicited strong proinflammatory responses, as measured by whole-fish cytokine expression and local activation of NF-κB signaling and TNF-α expression. This differential response is similar to what has been seen in epithelial cells *in vitro*, where many fungi activate NF-κB but only a challenge with C. albicans leads to further activation of inflammatory pathways and production of cytokines (16, 34, 35). Our results contrast with what is seen in phagocytes, which respond strongly *ex vivo* to both *Candida* species by producing proinflammatory cytokines (36, 38). One caveat to the work here, however, is that only single isolates of each species were tested in the zebrafish, and there are known isolate-specific differences in immune recognition and activation (90–96). It is intriguing that, in spite of the presence of phagocytes in both C. albicans and C. parapsilosis swimbladder infections, the signaling response *in vivo* to these mucosal infections is more similar to that for simplified *Candida*-human epithelium challenges than to that for *ex vivo Candida-*phagocyte challenges. C. parapsilosis supernatants have been shown to have an inhibitory effect on C. albicans-mediated invasion and damage to epithelial cells in coculture with C. albicans and on virulence in swimbladder infection; this may explain the lack of immune signaling in response to C. parapsilosis *in vivo* seen here (97). Our results are consistent with the idea that epithelial cells have a prominent role in regulating the overall inflammatory response to *Candida* at mucosal surfaces, in addition to acting as a physical barrier and initiating immune responses (98–101).

Using transgenic reporter zebrafish, we found differential patterns for the activation of NF-κB and expression of TNF-α in the swimbladder during C. albicans infection. NF-κB activation alone was seen in the epithelial layer surrounding the swimbladder, although both NF-κB activation and TNF-α expression were observed in cells that were not part of the epithelial layer, including macrophages. This may mean that the activation of immune pathways results in different responses in different cell types; for example, in epithelial cells *in vitro*, NF-κB is activated but does not lead to cytokine production (102). Alternatively, these differences may result from the different receptors mediating C. albicans recognition in epithelial cells and phagocytes (8, 103, 104) or from cross talk among cell types as the infection progresses (9, 42). It is unlikely that this differential expression pattern is due to reporter line differences, as many cell types, including epithelial cells and innate immune cells, are capable of activating NF-κB and expressing TNF-α in these fish lines (44, 63, 64, 105–109). Nonetheless, because no reporter gene completely recapitulates the activity of the native locus, these results should be extended through experiments using complementary reporters and reagents to test native expression patterns. Work with transgenic reporters for other signaling components, such as interleukin-1 (IL-1) (110), could contribute to deciphering this puzzle.

Phagocyte recruitment and activation are often associated with proinflammatory cytokine and chemokine production, but we observed recruitment and active engagement of both macrophages and neutrophils without significant cytokine elicitation in C. parapsilosis infection (111–113). Several noncytokine chemoattractants, such as reactive oxygen species, lipids, and secreted fungal molecules, are associated with fungal infection in mouse and zebrafish infection models (12, 75, 114–120). Thus, phagocyte recruitment in C. parapsilosis infection may be the result of noncytokine signals, underlining the potential importance of these alternative chemoattractants.

Although C. albicans and C. parapsilosis are two of the most common causes of systemic fungal infections, the risk factors for the two species differ. In humans, neutropenia is a major risk factor for disseminated C. albicans infection, but only a small percentage of C. parapsilosis cases involve neutrophil depletion (5, 83). Likewise, immunosuppressed mice are highly susceptible to C. albicans but not C. parapsilosis disseminated infection (121, 122). These differences are reflected in the experiments presented here, which show that neutrophils are not required for immunity to C. parapsilosis infection, in contrast to the previous finding that neutrophils are essential for protection from C. albicans mucosal infection (27). This difference may indicate that neutrophils are important in controlling hyphal growth of C. albicans but redundant for managing C. parapsilosis, whose yeast-only morphology may be contained by the remaining phagocytes (27, 123). Indeed, in the zebrafish, neutrophils and macrophages interacted with both hyphae and yeast of C. albicans, consistent with results from *in vitro* neutrophil and macrophage challenges (124–126). C. parapsilosis yeast and pseudohyphae are readily engulfed and killed by phagocytes *in vitro*, while engulfment of C. albicans requires longer times that vary with hyphal size and orientation (127–132). Although macrophages are known to provide protection from disseminated candidiasis, our recent work and that of others indicate that macrophages are redundant with respect to protection from mucosal C. albicans infection (23, 26, 27). In our higher-dose model, macrophages were recruited in significant numbers, activated NF-κB, expressed TNF-α, and interacted with both *Candida* species. It is intriguing that macrophages upregulate TNF-α upon C. albicans but not C. parapsilosis infection, suggesting that epithelium-macrophage cross talk or damage-induced signaling regulates cytokine production.

Overall, our work points to the unique characteristics of the zebrafish model (ease of live imaging and availability of transgenic lines) for discovery of previously unattainable information about host-pathogen interactions *in vivo*. Our comparison of host responses to two *Candida* species indicates that, unlike C. albicans, C. parapsilosis does not cause strong inflammatory responses or invasive disease at this mucosal site. We found a disconnect between inflammatory responses and phagocyte recruitment/activity that emphasizes the need for further study of signaling molecules that act on innate immune cells. Finally, imaging of single-cell patterns of gene activation paints a more complex picture of cell type-specific signaling during mucosal candidiasis. In sum, the tissue-specific aspects of the host response against *Candida* species are important and understudied aspects of disease that will benefit from future studies in zebrafish, mammalian hosts, and more complex *in vitro* challenge systems with more cell types.

## MATERIALS AND METHODS

### Candida strains and growth conditions.

*Candida* strains used in this study are listed in Table S1 in the supplemental material. *Candida* was maintained in YPD medium (20 g/liter peptone, 10 g/liter yeast extract; Difco) containing 2% glucose and glycerol (30%) at −80°C and then grown on YPD agar plates at 30°C. Single colonies were picked into 5 ml YPD liquid and grown at 30°C overnight on a rotator wheel (New Brunswick Scientific). Prior to injection into zebrafish swimbladders, *Candida* cultures were washed three times in phosphate-buffered saline (PBS), counted on a hemocytometer, and resuspended in 5% polyvinylpyrrolidone (PVP) (Sigma-Aldrich) in PBS at a concentration of 5 × 10^7^ cells/ml.

10.1128/mBio.00266-19.2 TABLE S1 *Candida* strains used in this study. Download Table S1, PDF file, 0.02 MB. Copyright © 2019 Archambault et al. 2019 Archambault et al. This content is distributed under the terms of the Creative Commons Attribution 4.0 International license.

### Animal care and maintenance.

Adult zebrafish were held in recirculating systems (Aquatic Habitats) at the University of Maine Zebrafish Facility, under a 14-h/10-h light/dark cycle and a water temperature of 28°C; they were fed Hikari micropellets (catalogue number HK40; Pentair Aquatic Ecosystems). Zebrafish strains used in this study are described in Table S2. Spawned eggs were collected and reared to 4 days postfertilization (dpf) at 33°C in E3 (5 mM sodium chloride, 0.174 mM potassium chloride, 0.33 mM calcium chloride, 0.332 mM magnesium sulfate, and 2 mM HEPES in Nanopure water [pH 7]) supplemented with 0.02 mg/ml of 1-phenyl-2-thiourea (PTU) (Sigma-Aldrich, St. Louis, MO) to prevent pigmentation. A temperature of 33°C was chosen as an intermediate temperature between the typical laboratory environment for zebrafish (28°C) and temperatures found in mouse and human (30°C on skin to 37°C core [133, 134]). We note that although temperature is a cue used by C. albicans to control morphology, other *in vivo* signals drive strong hyphal growth in the zebrafish, even at 28°C (84). When using D57N zebrafish, heterozygous transgenic fish were crossed with opposite-sex AB fish, and progeny were sorted for the presence of mCherry in neutrophils (D57N) or its absence (WT siblings). To obtain heterozygous offspring with consistent fluorescence levels, *Tg*(*NF-*κ*B*:*EGFP*) or *TgBAC*(*tnfa*:*GFP*) fish were crossed with opposite-sex AB fish, and embryos were screened on a Zeiss AxioVision VivaTome microscope (Carl Zeiss Microscopy, LLC) for basal GFP expression before injection. *mpeg1*:*GAL4/UAS*:*nfsB-mCherry* embryos were obtained by crossing *Tg*(*mpeg1*:*GAL4*):*gl24Tg* (65) fish with opposite-sex *Tg*(*UAS-E1b*:*NTR-mCherry*):*c264Tg* (66) fish.

10.1128/mBio.00266-19.3 TABLE S2 Zebrafish lines used in this study. Download Table S2, PDF file, 0.02 MB. Copyright © 2019 Archambault et al. 2019 Archambault et al. This content is distributed under the terms of the Creative Commons Attribution 4.0 International license.

### Zebrafish infections.

Zebrafish infections were carried out by glass needle injection into the swimbladder as previously described (55). Briefly, zebrafish at 4 dpf were anaesthetized with Tris-buffered tricaine methane sulfonate (160 μg/ml) (Tricaine; Western Chemicals, Inc., Ferndale, WA) and injected with 4 nl PVP alone or PVP containing 5 × 10^7^ yeast cells/ml of C. albicans or C. parapsilosis. Infected fish were placed in individual wells of a 96-well glass-bottom imaging dish (Greiner Bio-One, Monroe, NC) and screened for an inoculum of 50 to 100 yeast cells on a Zeiss AxioVision VivaTome microscope. For survival curves, injected fish that passed screening were held for 4 days postinjection and monitored daily for survival.

### Fluorescence microscopy.

For imaging, fish were anaesthetized with Tricaine, immobilized in 0.5% low-melting-point agarose (Lonza, Switzerland) in E3 containing Tricaine, and arranged in a 96-well glass-bottom imaging plate. Images were made on an Olympus IX-81 inverted microscope with an FV-1000 laser scanning confocal system (Olympus, Waltham, MA), using a 20×/0.7-numerical-aperture (NA) or a 10×/0.4-NA lens objective. EGFP, dTomato/mCherry, and infrared fluorescent proteins were detected by laser/optical filters for excitation/emission at 488 nm/505 to 525 nm, 543 nm/560 to 620 nm, and 635 nm/655 to 755 nm, respectively. Images were collected with FluoView (Olympus) software.

### Dissected swimbladders.

After live imaging, chosen zebrafish were euthanized with a Tricaine overdose at 25 to 27 hpi, and swimbladders were removed with fine forceps as described previously (55). Swimbladders were transferred to 0.4% low-melting-point agarose in PBS on a 25- by 75- by 1.0-mm microscope slide and covered with an 18- by 18-mm no. 1.5 coverslip. Preapplied dabs of high-vacuum grease (Dow Corning, Midland, MI) at the corners of the coverslip prevented crushing and deflation of the swimbladder. The slides were imaged within 15 min on an Olympus IX-81 inverted confocal microscope using a 20×/0.7-NA lens objective as described above.

### Quantitative real-time PCR.

Total RNA was extracted by homogenizing groups of 10 to 14 whole, euthanized larvae in TRIzol (Invitrogen, Carlsbad, CA). Cleanup was achieved using an RNeasy kit (Qiagen, Germantown, MD) according to the manufacturer’s protocol, with the addition of an on-column DNase step (New England BioLabs, Ipswich, MA). RNA was eluted in 20 μl of nuclease-free water and stored at −80°C. cDNA was synthesized from 500 ng of RNA per sample using iScript reverse transcription (RT) supermix for RT-qPCR (Bio-Rad, Hercules, CA), and a no-RT reaction was performed for each sample. qPCR was carried out using SsoAdvanced universal SYBR green supermix (Bio-Rad), in 10-μl reaction mixtures, using 1 μl cDNA per reaction and a 0.3 μM primer concentration, on a CFX96 thermocycler (Bio-Rad). Threshold cycle (*C_T_*) values and dissociation curves were analyzed with Bio-Rad CFX Manager software. The change in gene expression was normalized to the *gapdh* level (Δ*C_T_*) and then compared to the value for vehicle-injected controls (ΔΔ*C_T_*) using the 2^−ΔΔ*C_T_*^ method (135). Primers (Integrated DNA Technologies) are listed in Table S3.

10.1128/mBio.00266-19.4 TABLE S3 qPCR primer information. Download Table S3, PDF file, 0.02 MB. Copyright © 2019 Archambault et al. 2019 Archambault et al. This content is distributed under the terms of the Creative Commons Attribution 4.0 International license.

### Image analysis.

The percentage of the swimbladder covered by *Candida* at 24 hpi was determined using Fiji software (ImageJ environment [136]) applied to maximum-projection images from stacks of 15 to 25 z-slices. Images were taken with identical acquisition settings to ensure comparability. The swimbladder area was delineated, and the percent coverage of *Candida* fluorescence above a set threshold (corresponding to background fluorescence) was calculated. Images of the swimbladder areas of *Tg*(*NF-*κ*B*:*EGFP*) and *TgBAC*(*tnfa*:*GFP*) fish were analyzed using Fiji software. Images covered the swimbladder from midline to skin in 5-μm z-slices. The number of slices per image ranged from 12 to 22, depending on the size of the fish. Time-lapse images were processed in Fiji using descriptor-based registration (137). Neutrophils and macrophages were outlined and counted in FluoView (Olympus), from images taken at 24 hpi.

### Statistical analysis.

Statistical analyses were carried out using GraphPad Prism 7 software (GraphPad Software, Inc., La Jolla, CA). All significant differences are indicated in the figures. When data failed to pass the D’Agostino-Pearson test for normal distribution of data, or when the number of samples was too small to determine normality, nonparametric statistics were used (Fig. 1B, Fig. 2, Fig. 3D and E, Fig. 5H, Fig. 6D and E, and Fig. 7B). Kaplan-Meier survival curves were subjected to a log rank (Mantel-Cox) test, and Bonferroni correction was then used to determine statistical differences between pairs of treatments (Fig. 1G and Fig. 7A). NF-κB activation, TNF-α expression, macrophage recruitment, and qPCR results were analyzed using the Kruskal-Wallis test by ranks and Dunn’s test for multiple comparisons (Fig. 2, Fig. 3D and E, Fig. 6D, and Fig. 7B). Neutrophil recruitment data were normally distributed, so analysis of variance (ANOVA) with Tukey’s test for multiple comparisons was used (Fig. 5D). To compare *Candida* burdens and phagocyte interactions, we used the Mann-Whitney test (Fig. 1B, Fig. 5H, and Fig. 6E). Fisher’s exact test was used to compare the neutrophils and macrophages engaged in phagocytosis of the two *Candida* species (Fig. 5I and Fig. 6F). Paired *t* tests were used to compare interactions of phagocytes with C. albicans hyphae and yeast (Fig. 5J and Fig. 6G).

### Ethics statement.

All zebrafish studies were carried out in accordance with the recommendations in the *Guide for the Care and Use of Laboratory Animals* of the National Institutes of Health (138). All animals were treated in a humane manner and euthanized with Tricaine overdose according to guidelines of the University of Maine IACUC, as detailed in protocol number A2015-11-03.
