# Supplementary figures and images for: Intravital Imaging Reveals Divergent Cytokine and Cellular Immune Responses to Candida albicans and Candida parapsilosis
Source: mBio. 2019 May 14;10(3):e00266-19. doi: 10.1128/mBio.00266-19 (PMC6520444; doi:10.1128/mBio.00266-19)

**S1**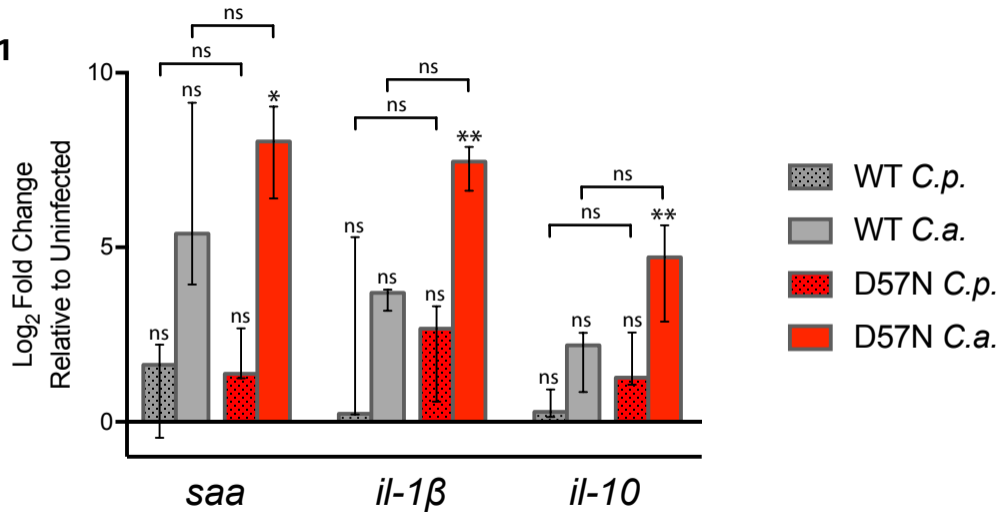

Supplement: FIG S1 [file mBio.00266-19-sf001.pdf]
