# Supplementary material for: Intravital Imaging Reveals Divergent Cytokine and Cellular Immune Responses to Candida albicans and Candida parapsilosis
Source: mBio. 2019 May 14;10(3):e00266-19. doi: 10.1128/mBio.00266-19 (PMC6520444; doi:10.1128/mBio.00266-19)
Supplement: TABLE S1 [file mBio.00266-19-st001.pdf]

Table S1. *Candida* strains used in this study.

Strain	Source/Reference
C. albicans SC5314 Caf2.1-dTom-NATr	R. L. Gratacap, J. F. Rawls, and R. T. Wheeler, *Dis. Model. Mech.* 6: 1260–70, 2013, doi: 10.1242/dmm.012039
C. albicans WT-GFP (SC5314 (Peno1-yEGFP$_3$-NAT))	R. T. Wheeler, D. Kombe, S. D. Agarwala, and G. R. Fink, *PLoS Pathog.* 4: 1–12, 2008, doi: 10.1371/journal.ppat.1000227
C. albicans SC5314 Caf2:FR	A. C. Bergeron, B. G. Seman, J. H. Hammond, L. S. Archambault, D. A. Hogan, and R. T. Wheeler, *Infect. Immun.*, 2017, doi:10.1128/IAI.00475-17
C. parapsilosis clinical isolate 4175 (A010) CpURA3/CpURA3 ENO1/ENO1::GFP-NAT1	S. Gonia, B. Larson, and C. A. Gale, *Yeast*, 33(2): 63–9, 2016, doi: 10.1002/yea.3141
C. parapsilosis clinical isolate 4175 (A010) CpURA3/CpURA3 ENO1-mCherry-NAT1/ENO1	
