# Supplementary material for: Intravital Imaging Reveals Divergent Cytokine and Cellular Immune Responses to Candida albicans and Candida parapsilosis
Source: mBio. 2019 May 14;10(3):e00266-19. doi: 10.1128/mBio.00266-19 (PMC6520444; doi:10.1128/mBio.00266-19)
Supplement: TABLE S2 [file mBio.00266-19-st002.pdf]

Table S2. Zebrafish lines used in this study.

Strain	Source/Reference
Wildtype AB	Zebrafish International Resource Center (ZIRC)
Tg(mpx:GFP)i114Tg	S. Renshaw and C. Loynes, *Blood* 108:3976–3978, 2006, doi: 10.1182/blood-2006-05-024075
Tg(mpx:mCherry)uwm7Tg	S. K. Yoo, Q. Deng, P. J. Cavnar, Y. I. Wu, K. M. Hahn, and A. Huttenlocher, *Dev. Cell* 18: 226–236, 2010, doi: 10.1016/j.devcel.2009.11.015
Tg(mpeg1:GAL4)gl24Tg	F. Ellett, L. Pase, J. W. Hayman, A. Andrianopoulos, and G. J. Lieschke, *Blood*, 117:e49-56, 2011, doi: 10.1182/blood-2010-10-314120.
Tg(UAS-E1b:NTR-mCherry)c264Tg	M. G. Goll, R. Anderson, D. Y. R. Stainier, A. C. Spradling, and M. E. Halpern, *Genetics*, 182: 747–755, 2009, doi: 10.1534/genetics.109.102079
Tg(mpeg1:dTomato)	A. J. Pagan *et al.*, *Cell Host Microbe*, 18: 15–26, 2015, doi: 10.1016/j.chom.2015.06.008
TgBAC(tnfa:GFP)pd1028Tg	L. Marjoram *et al.*, *Proc. Natl. Acad. Sci.*, 112: 201424089, 2015, doi: 10.1073/pnas.1424089112
Tg(6xHsa.NFKBN:EGFP)nc1Tg	M. Kanther *et al.*, *Gastroenterology*, 141: 197–207, 2011, doi: 10.1053/j.gastro.2011.03.042
Tg(mpx:mCherry,rac2_D57N)zf307Tg	Q. Deng, S. K. Yoo, P. J. Cavnar, J. M. Green, and A. Huttenlocher, *Dev. Cell*, 21: 735–45, 2011, doi: 10.1016/j.devcel.2011.07.013
