# Supplementary material for: Intravital Imaging Reveals Divergent Cytokine and Cellular Immune Responses to Candida albicans and Candida parapsilosis
Source: mBio. 2019 May 14;10(3):e00266-19. doi: 10.1128/mBio.00266-19 (PMC6520444; doi:10.1128/mBio.00266-19)
Supplement: TABLE S3 [file mBio.00266-19-st003.pdf]

Table S3. qPCR primer information

Gene	Sequence (5'-3')	Reference
ccl2	Fw, GTCTGGTGCTCTTCGCTTTC<br>Rv, TGCAGAGAAGATGCGTCGTA	C. J. Cambier *et al.*, *Nature*: 505:218–22, 2014, doi: 10.1038/nature12799
cxcl8	Fw, TGCATTGAAACAGAAAGCCGACG<br>Rv, ATCTCCTGTCCAGTTGTCATCAAGG	A. C. Bergeron, B. G. Seman, J. H. Hammond, L. S. Archambault, D. A. Hogan, and R. T. Wheeler, *Infect. Immun.*, p. IAI.00475-17, 2017, doi:10.1128/IAI.00475-17
il6	Fw, GGACGTGAAGACACTCAGAGACG<br>Rv, AAGGTTTGAGGAGAGGAGTGCTG	
il10	Fw, ATTTGTGGAGGGCTTTCCTT<br>Rv, AGAGCTGTTGGCAGAATGGT	F. J. Roca *et al.*, *J. Immunol.*, 181: 5071–5081, 2008, doi: 10.4049/jimmunol.181.7.5071
saa	Fw, CGGGGTCCTGGGGGCTATTG<br>Rv, GTTGGGGTCTCCGCCGTTTC	B. Lin *et al.*, *Mol. Immunol.*, 44: 295–301, 2007, doi: 10.1016/J.MOLIMM.2006.03.001
tnfa	Fw, CGCATTTCACAAGCGAATTT<br>Rv, CTGGTCCTGGTCATCTCTCC	R. L. Gratacap, J. F. Rawls, and R. T. Wheeler, *Dis. Model. Mech.* 6: 1260–70, 2013, doi: 10.1242/dmm.012039
gapdh	Fw, TGGGCCCATGAAAGGAAT<br>Rv, ACCAGCGTCAAAGATGGATG	C. J. Mattingly, T. H. Hampton, K. M. Brothers, N. E. Griffin, and A. Planchart, *Environ. Health Perspect.*, 117: 981–7, 2009, doi: 10.1289/ehp.0900555
